# Direct Endoscopic Necrosectomy: Timing and Technique

**DOI:** 10.3390/medicina57121305

**Published:** 2021-11-28

**Authors:** Sergio Pinto, Saverio Bellizzi, Roberta Badas, Maria Laura Canfora, Erica Loddo, Simone Spada, Kareem Khalaf, Alessandro Fugazza, Silvio Bergamini

**Affiliations:** 1 Digestive Endoscopy Unit, Department of Surgical Sciences, University Hospital of Cagliari, 09042 Cagliari, Italy; rbadas@aoucagliari.it (R.B.); mcanfora@aoucagliari.it (M.L.C.); ery.loddo@gmail.com (E.L.); s.spada@aoucagliari.it (S.S.); s.bergamini@aoucagliari.it (S.B.); 2 Medical Epidemiologist, Independent Consultant, 1202 Geneva, Switzerland; saverio.bellizzi@gmail.com; 3 Department of Biomedical Sciences, Humanitas University, Pieve Emanuele, 20090 Milano, Italy; kareem.khalaf@st.hunimed.eu; 4 Digestive Endoscopy Unit, Department of Gastroenterology, Humanitas Research Hospital-IRCCS, 20089 Rozzano, Italy; alessandro.fugazza@humanitas.it

**Keywords:** walled-off pancreatic necrosis, pancreatic necrotic collection, acute pancreatitis, direct endoscopic necrosectomy, EUS, LAMS

## Abstract

Walled-off pancreatic necrosis (WOPN) is one of the local complications of acute pancreatitis (AP). Several interventional techniques have been developed over the last few years. The purpose of this narrative review is to explore such methodologies, with specific focus on endoscopic drainage and direct endoscopic necrosectomy (DEN), through evaluation of their indications and timing for intervention. Findings indicated how, after the introduction of lumen-apposing metal stents (LAMS), DEN is becoming the favorite technique to treat WOPN, especially when large solid debris or infection are present. Additionally, DEN is associated with a lower adverse events rate and hospital stay, and with improved clinical outcome.

## 1. Introduction

Acute pancreatitis (AP) represents one of the most common gastroenterological disorders requiring hospitalization [1,2].

Most cases of AP undergo an uncomplicated course, where supportive care with intravenous fluid infusion, nutritional support, analgesic therapy, and accurate monitoring suffice to achieve a full recovery [2,3]. Yadav and Lowenfels reported a worldwide incidence of AP between 13 and 45 cases per 100,000 persons per year [4].

According to the 2012 revised Atlanta classification and definitions [1], AP can be subdivided in two types, which based on its appearance on contrast-enhanced computed tomography (CECT) [5]. The first is interstitial oedematous pancreatitis, which is the most common type, characterized by diffuse or localized pancreatic and peripancreatic inflammatory oedema, and necrotizing pancreatitis (5–10% of AP cases), characterized by the presence of pancreatic (<5%) or peripancreatic necrosis (about 20%). The second is both pancreatic and peripancreatic necrosis (75–80%) [6]; the aforementioned features an increased mortality rate up to 32% when a superimposed infection occurs, which happens in about one third of these patients [3,7]. Pancreatic fluid collections (PFCs), develop as local complications of AP in about 20% of cases [8].

Depending on the type of AP, PFCs can contain liquid or necrotic material, and are classified as either:

Acute peripancreatic fluid collections (APFCs): are retroperitoneal extra-pancreatic collections confined by the normal fascial planes, without a defined wall, which occurs in interstitial oedematous acute pancreatitis [1].

Pancreatic pseudocysts (PPCs): are retroperitoneal peripancreatic or, occasionally, intrapancreatic fluid collections, confined by a defined wall composed of granulation tissue, fibrous tissue, and blood vessels, which contain homogeneous sterile amylase rich fluid with no solid debris and which also occur in interstitial oedematous acute pancreatitis [1,9].

Acute necrotic collections (ANCs): are pancreatic and/or peripancreatic collections of inhomogeneous fluid and necrotic material, without a defined wall [9]. They may be multiple and multiloculated and arise within the first 4 weeks of acute necrotizing pancreatitis (ANP) from the liquefaction of solid necrotic areas. ANCs may gradually resolve spontaneously, get infected (30% of cases) or evolve into walled-off necrosis [5,10].

Walled-off pancreatic necrosis (WOPN): are pancreatic and/or peripancreatic collections of necrotic tissue, which arise from the maturation of ANCs. Within approximately 2–4 weeks or more, a capsule made of granulation tissue and collagen forms. This might be a natural defense against the spreading of pancreatic enzymes, inflammation, and necrosis, which are contained by the fibrous wall [11,12]. It may be single or multiple and carries a higher risk of infection [9,13].

**Figure 1:**
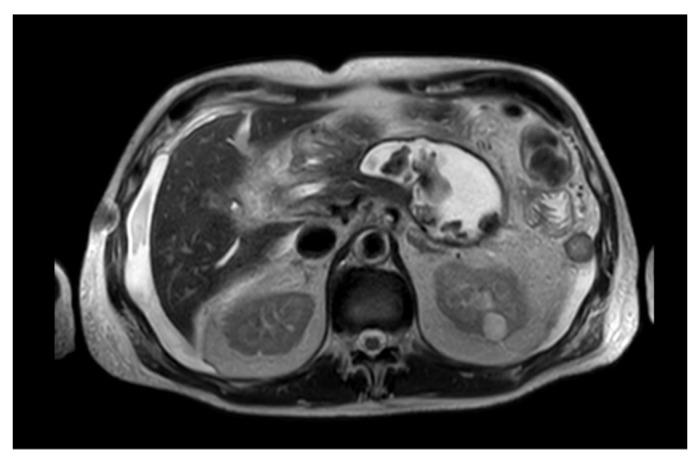
MRI image of a WOPN with both liquid and solid necrotic material within.

Due to their higher morbidity and mortality, pancreatic necrotic collections (PNCs) need to be promptly recognized and adequately treated [14]. Therefore, distinguishing between a necrotic and a fluid collection is crucial to guarantee the best outcome for the patient. Differentiating between a PPC and a WOPN may not always be obvious under CECT; MRI and endoscopic ultrasonography (EUS) evaluation are better tools to identify solid debris inside the collection (Figure 1) [1,15].

### 1.1. Indication for Intervention in PNCs

In about half of the WOPN cases, patients are completely asymptomatic; whilst the other half may be burdened with malaise, abdominal pain, anorexia, weight loss, relapsing or recurrent pancreatitis, fever (in case of infection), compression or erosion and fistulization in the adjacent structures (stomach, blood vessels, bile duct) [11,16,17]. According to the current international guidelines, WOPN requires intervention only when patients become infected, such as the case of persistent organ failure or failure to thrive, or the case of adjacent organ compression or persistent symptoms, even if it’s sterile [2,13,14].

Infection complicates about 30% of ANP cases and is associated with an increased mortality rate (up to 32%, versus 10% of sterile ANP) [3,7,13], requiring a prompt diagnosis to organize an appropriate therapeutical strategy. A combination between clinical signs (fever, bacteriemia, worsening leukocytosis, new/prolonged organ failure, increasing or elevated CRP and procalcitonin levels), radiological signs (gas bubbles in a necrotic collection at the CECT), fine needle aspiration (FNA) and subsequent GRAM stain or culture, seems to be the best strategy to diagnose ANP. Gas bubbles are seen at CECT in only half of the patients with ANP, and FNA has a false negative rate of 29% [12,17]. Thus, routine FNA of the necrotic collection is not advised and it may be performed in case of no benefit from the initial antibiotic therapy, or when clinical and radiological signs are unclear [14,17].

There is no evidence to support a broad spectrum intravenous antibiotic prophylaxis for the prevention of infection in ANP [2]. Such therapy should be started only when infection is suspected; for this purpose, carbapenems, quinolones, metronidazole, and third- or higher-generation cephalosporins showed a good penetration in the pancreatic and peripancreatic tissue [2,13]. Since less than 5% of patients with infected pancreatic necrosis recover with antibiotic therapy alone, it is almost always an indication for invasive intervention [12].

### 1.2. Evolution of Interventional Techniques: From the Operating Theatre to the Endoscopic Room, a Target Approach

Many interventional strategies have been proposed to treat PNCs. Over the years we assisted to a gradual transition from traditional open surgery to minimally invasive surgery and endoscopic interventions.

The current international guidelines suggest adopting the so called “step-up approach” indicating a gradual increase from a less invasive to a more invasive procedure, when needed [2,13,14]. This concept may seem obvious and reasonable, but many studies were necessary to reach such an agreement, and more are still needed to further define the best way to treat PNCs. Furthermore, considering the broad diversity of each PNC (anatomical site, number, dimension, percentage of solid debris, infection, involvement of contiguous structures, amount of defined wall and its thickness, presence of wall blood vessels) together with the diversity of the clinical condition of each patient, trying to find a single technique to treat all the PNCs, may be unreasonable. In this scenario, targeting the best procedure for each patient is crucial. Until 2010, the standard approach to symptomatic or infected PNC was open surgery: laparotomic debridement of the necrotic tissue and subsequent placement of drainage and lavage tubes [14]. In order to reduce mortality, risk of iatrogenic infection and systemic inflammation, intervention should be postponed for 4 weeks after the onset of symptoms, allowing enough time for the collection to be completely encapsulated (i.e., WOPN) [18,19]. Despite such precautional measures, open necrosectomy is associated with a high rate of local and systemic complications (35 to 95%) and death (11 to 39%) [20,21,22]. Therefore, with the evolution of technology and techniques, new strategies of intervention have been evaluated to reach better outcomes.

The PANTER trial published in 2010, represents a milestone for the WOPN surgical treatment [22]. Van Santvoort et al. demonstrated that a minimally invasive step-up surgical approach (percutaneous drainage followed, if necessary, by a video-assisted retroperitoneal debridement (VARD), and subsequent lavage of the cavity through percutaneous drainage) on patients with infected WOPN was superior to open necrosectomy in terms of new onset organ failure and diabetes, necessity of ICU admission, incisional hernias and total cost, with similar mortality rate [22].

In line with this minimally invasive approach, endoscopists started performing drainage of PFCs through the stomach or duodenal wall.

The first pioneering direct endoscopic necrosectomy (DEN) was described in 2000 by Seifert et al. on three patients with infected WOPN, not fit enough for a surgical intervention [23]. They inserted a gastroscope directly into the cavity and performed the debridement of the necrotic material using a stone-retrieval basket.

The term “direct” refers to the access to the necrotic collection gained directly by the endoscope through the gastric or duodenal wall, allowing visualization and removal of necrotic debris [24]. Small DEN series, conducted mainly in Europe, showed encouraging results in infected and symptomatic WOPN [25,26,27,28].

In order to assess DEN efficacy, complications and mortality rate, Seifert et al. carried a large retrospective multicenter study with long-term follow-up (the GEPARD study) [24]. A sample of 93 patients with infected WOPN containing >50% of solid debris on ultrasonography or EUS, underwent DEN after a mean of 41 days (4–158) after AP onset. A mean of 6.2 DEN sessions (1–35) were conducted every 1–4 days until all debris and necrotic material were removed. A minimum of 2 years follow-up (clinical, laboratory and imaging) was conducted, showing an initial success rate of 81%, a long-term clinical efficacy rate of 68%, a complication rate of 26% and a procedure-related mortality rate of 7.5% [24].

A study by Gardner et al. on 104 patients with infected or symptomatic WOPN reached even better clinical outcomes than the GEPARD study [29]. Resolution of the collection was achieved in 91.3% of patients, with a periprocedural adverse events (AEs) rate of 14% and a mortality rate of 5.7%.

It seemed clear that DEN was a feasible and less invasive alternative to surgery for treating WOPN. However, lack of comparative trials made it difficult to definitely assess the real effectiveness and safety of the DEN compared to other techniques. A multicenter randomized controlled trial (the PENGUIN trial [30]) conducted by Bakker et al. attempted to fill that gap. They compared the proinflammatory response and the clinical outcome between DEN and surgical necrosectomy (VARD or open, preceded by percutaneous drainage of the collection) in patients with infected WOPN [30]. According to their results, DEN was associated with significantly lower IL-6 levels (which is a pro-inflammatory cytokine) and lower rate of complications (20% vs. 80%).

The Dutch Pancreatitis Study Group conducted a randomized trial (the TENSION trial) to investigate whether an endoscopic step-up approach (EUS-assisted transgastric or transduodenal placement of two double pigtail stents and a nasocystic catheter; if no clinical improvement was achieved, one or more DEN was performed) was superior to the surgical step-up approach (CT-guided or ultrasound-guided percutaneous catheter drainage; if no clinical improvement was succeeded, a VARD procedure was performed) in patients with infected pancreatic collection [31]. Despite comparable results in terms of major complications and mortality rates between the two groups, cardiovascular and persistent cardiovascular organ failure were lower in the endoscopy group, as was the incidence of pancreatic fistulas (with subsequent increase in hospital stay and follow up evaluation), number of drainage catheter repositionings and hospital stay. Moreover, in 2018, a pooled analysis conducted on 1980 severely ill patients with ANP who underwent necrosectomy, found that minimally invasive surgical necrosectomy and DEN were associated with reduced death rates compared with open necrosectomy [21].

Two recent meta-analyses independently conducted by Bang et al. and Haney et al. [32,33] on the same three randomized trials involving 184 patients with infected WOPN [30,31,34], compared clinical outcomes of minimally invasive surgical intervention and endoscopic intervention. Endoscopic intervention was associated with less new onset organ failure, enterocutaneous fistula or perforation of a visceral organ, pancreatic fistula and shorter hospital/ICU stay.

Another recent meta-analysis conducted by Khan et al. [35] on two randomized trials and four observational studies, involving 641 patients with infected or symptomatic WOPN compared safety and clinical outcomes of minimally invasive surgical intervention to endoscopic intervention. Endoscopic intervention was associated with lower mortality, less new onset organ failure, enterocutaneous fistula or perforation of a visceral organ, pancreatic fistula and shorter hospital/ICU stay.

These studies combined suggest that an endoscopic step-up approach, when possible, is the most advisable procedure to treat patients with infected WOPN in order to reduce complications and mortality rates, hospital stay and total costs. If the necrotic collection is not reachable endoscopically (when involving one or both the paracolic gutters and/or the pelvis) or if the endoscopic approach is not available or unsuccessful, a mini-invasive surgical step-up approach is recommended [2,13,14].

### 1.3. Endoscopic Step-Up Approach

The aim of an endoscopic intervention on symptomatic or infected WOPN is to gain access to the collection and perform a transmural drainage of the content into the gastric/duodenal lumen, using plastic or metal stents. Necrosectomy may be performed using the already finished fistulas and, depending on the kind of stent used, through the stent itself.

#### 1.3.1. Double Pigtail Plastic Stent Drainage

Drainage can be obtained by EUS-guided single or multiple cystenterostomy, positioning one (single transluminal gateway technique, STGT) or more (multiple transluminal gateway technique, MTGT) double pigtail plastic stents (DPPs) through the stomas [13,36]. MTGT, firstly performed by Varadarajulu et al. in 2011, should be used as a first step on those patients with multiple or large (>12 cm) WOPN, where a STGT may be ineffective, or as a second step after suboptimal drainage with previous STGT [13,36]. Data about WOPN resolution with DPPS drainage alone, show a clinical success between 30.8% and 52.1% [31,36,37].

#### 1.3.2. Fully Covered Self-Expandable Metal Stents

DPPSs are notoriously cheap and widely diffused; but their small 7–10 F caliber does not allow a good drainage of the larger solid necrotic debris, with frequent stent lumen occlusion. Moreover, they require multiple stent placement and dilation procedures to obtain a cystoenterostomy of caliber wide enough to perform DEN [38]. Fully covered self-expandable metal stents (FCSEMSs) represented the first alternative to DPPSs. Their larger diameter allows better drainage of solid debris, with lower rates of occlusion [9,37,38,39].

Biliary stents are successfully used to drain PFCs; however, their small diameter is not sufficient to avoid the occlusion of the lumen by solid necrotic material and does not allow passage of the endoscope to perform DEN through the stent itself [9,13,38]. Esophageal FCSEMSs have been first used to treat WOPN endoscopically in 2009 by Antillon et al., who successfully treated a patient with infected WOPN after four inefficacious DEN sessions, demonstrating that the use of a 22 mm FCSEMS with intensive lavage may be an effective option to facilitate WOPN drainage in selected patients [40]. Sarkaria et al. reported their experience with 18–23 mm esophageal FCSEMSs on 17 patients with infected WOPN, with an overall success rate of 83%, a faster stent deployment compared to DPPS, and no need to replace the stent between each session [38].

The main AEs using FCSEMS are: stent migration, bleeding from the fistulous tract, stent migration and injury of the duodenal/gastric wall or of the retroperitoneum, resulting in bleeding and/or perforation [9,41].

#### 1.3.3. Lumen-Apposing Metals Stents (LAMSs)

##### Technical Aspects of LAMSs

To overcome DPPS and FCSEMS limitations, in 2011 a different kind of SEMS was developed, namely lumen apposing metal stent (LAMS) [42]. The AXIOS stent (Boston Scientific, Marlborough, MA, USA) was the first LAMS commercialized. It is made of braided nitinol, fully covered with silicone, with a dog bone shape given by two bilateral double-walled anchoring flanges with a 90° angle between each flange and the narrower central part of the stent. Its design should prevent stent migration, allowing the apposition of the walls of two luminal structures, putting them in communication and preventing leakage from the fistula. Its delivery method consists of a multistep EUS-guided procedure: using a through-the-scope device (compatible with therapeutic echoendoscopes with a ≥3.7-mm working channel) [43].

After the creation of a fistula between the two luminal structures by needle plus guidewire and its dilation with a pneumatic balloon over a guidewire, the LAMS is progressively deployed [42]. After AXIOS introduction, different manufacturers produced different kinds of LAMSs and SEMS with anti-migratory systems with different features and delivery mechanisms (Spaxus and Nagi, by Taewoong Medical, Gimposi, Korea; Aixstent, by Leufen Medical, Berlin, Germany; Hanarostent Plumber, by M.I. Tech, Pyeongtaek-si, Korea) [44,45].

An evolution of this device was introduced in 2015 by Boston Scientific: an electrocautery-LAMS (EC-LAMS) (HOT-AXIOS, Boston Scientific, Marlborough, MA, USA). This represents a second-generation device, implemented with a cautery tip for the fistula creation. The new system simplifies the delivery of the LAMS by immediate one-step procedure, without the need of an access for needle plus guidewire, or further dilation of the fistula [41].

**Figure 2:**
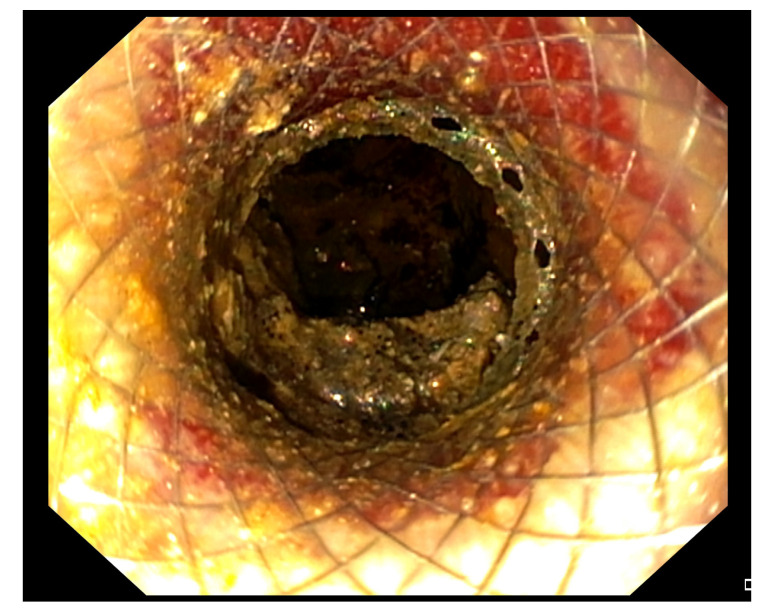
Endoscopic view of a WOPN cavity through the lumen of the LAMS, draining necrotic material.

The EC-LAMS is available in many different diameters and lengths to better adapt to anatomical sites (6 × 8 mm, 8 × 8 mm, 10 × 10 mm, 15 × 10 mm, 15 × 15 mm, and 20 × 10 mm). Their larger diameter and shorter length, together with the peculiar shape and the delivery system, should provide a better drainage of necrotic solid debris, reducing the risk of migration, perforation, and intra-operative and post-operative bleeding [46,47,48,49]. Furthermore, it allows access through its lumen to the WOPN by an endoscope to perform DEN (Figure 2).

##### Clinical Practice with LAMSs

After LAMS diffusion, many studies have been conducted in the attempt to assess its efficacy, safety, and AEs rate in WOPN treatment.

Both LAMS and EC-LAMS have shown high technical (95–100%) and clinical (84.2–93.9%) success rates [43,44,47,48,49]. Sharahia et al. reported a WOPN resolution after only drainage in 27.1% with symptomatic WOPN using a single 10 or 15 mm, showing a higher clinical success with the bigger LAMS [43].

Similarly, comparing the 15- and 20-mm EC-LAMS, Parsa et al. reported that fewer DEN sessions are needed to reach WOPN resolution when using the 20 mm EC-LAMS [44] and Bekkali et al. found that the use of EC-LAMS was associated with a reduced procedure time when compared with a first-generation LAMS in drainage and DEN [50]. Binda et al. demonstrated how MTGT can also be safely and efficaciously carried out with EC-LAMS for treating complex WOPN [50].

In our clinical practice we usually place a large LAMS (15 or 20 mm in diameter) depending on the amount of necrotising of the WOPN. After LAMS deployment we dilate the stent with a pneumatic balloon to check its contents, the amount of necrosis and the presence of vessels. The large diameter of the LAMS facilitates drainage of necrotic contents leading to faster resolution of the collection. For this reason, DEN could be avoided in case of symptoms improvement. Then, a follow-up CECT scan to verify treatment response is organized at 3 weeks, followed by LAMS removal in case of WOPN resolution, as suggested by Bang et al. [51].

The overall LAMS-related AEs rate treating WOPN appears difficult to evaluate because of the lack of data uniformity among published series. The main AEs reported are migration/dislodgement of the stent (2.6–10.6%), occlusion (0.9–8.7%), bleeding (0.7–5.5%) and perforation (0–1.3%) [39].

Dislodgement of the LAMS, in particular during DEN, could be resolved with the replacement of the same stent [52].

On the contrary, bleeding and perforation are life threatening and challenging AEs, since they can involve both the enteric and the retroperitoneal wall. As previously mentioned, the higher diameter of LAMSs and its shorter length, compared to the dimensions of the other stents, should reduce the risk of bleeding both from the fistula (by compressing surrounding wall vessels) and from the enteric and retroperitoneal wall (due to its reduced protruding portion).

While this seems true for the fistular bleeding, this may not be correct for the retroperitoneal bleeding. During a comparative trial with EC-LAMS and DPPSs, Bang et al. noticed that a higher than anticipated procedural AEs rate occurred in the LAMS group (50% vs. 0%) [51]. The AEs included bleeding, buried stent syndrome and obstructive jaundice secondary to stent-induced biliary stricture. Since all of the AEs appeared after 3 weeks from LAMS positioning, authors hypothesized that the wide caliber of the stent may lead to a fast resolution of the WOPN with subsequent collapse of the wall of the collection and friction against the distal flange of the LAMS, and subsequent erosion, perforation, and bleeding (including pseudoaneurysm bleeding). For the same reason, since the LAMS is immobile through the gastric/duodenal wall, it can become deeply buried into the wall itself, leading to the “buried stent syndrome” or to fibrotic reaction involving adjacent structures, like the common bile duct. Thus, authors suggest performing a CT scan in 3 weeks, followed by removal of the LAMS if the WOPN has resolved [51]. Therefore, early LAMS removal after 4 weeks is becoming a widely suggested practice to avoid late AEs [53].

Data reported by Fugazza et al. on a large international cohort of 304 patients that underwent drainage of PFCs with LAMS, show how about 60% of bleeding episodes occurred in the first 14 days after LAMS positioning, raising attention to the fact that LAMS-related bleeding cannot be considered exclusively as a late AE (no protective role of EC-LAMS was observed for bleeding or other AEs) [54]. Furthermore, they showed how the pneumatic dilation of the LAMS after deployment reduces the risk of AEs.

#### 1.3.4. Comparing DPPSs with LAMS

These studies suggest that both LAMS and EC-LAMS are safe devices, with high technical and clinical success rates in WOPN treatment. The debate about safety and efficacy of the LAMS compared to the DPPS is still a controversial topic among the scientific community.

A recent systematic review and meta-analysis conducted on 30 studies with 1524 patients with WOPN, tried to solve such doubts [39]. Despite the already mentioned difficulty to perform such analysis because of the lack of uniformity in outcome definitions and reporting across studies, they found a similar AEs rate (bleeding, perforation, stent migration and stent occlusion) and similar efficacy rate between the two devices. Chen et al. conducted a cost-effectiveness analysis comparing LAMSs with DPPSs in the management of WOPN, reporting that, despite a higher cost of LAMSs, their higher efficacy (92% vs. 84%) makes this device more cost-effective [55].

#### 1.3.5. Endoscopic Gastric Fenestration (EGF)

With the purpose to overcome disadvantages of DPPs and LAMS for the therapy of WOPN (small caliber and poor drainage, occlusion of the stent, bleeding, migration, jaundice, and high cost) Liu et al. have recently explored a different modality to gain access to the necrotic collection, the endoscopic gastric fenestration (EGF) [56]. For the feasibility of the procedure, a complete evaluation of the characteristics of the collection with CECT and EUS is performed, as a close adherence between the gastric and the collection wall is mandatory to proceed. Total intravenous anesthesia and tracheal intubation are performed. EUS guidance guarantees to find and mark the best site on the gastric wall to perform a fenestration by endoscopic submucosal dissection (ESD), until the muscularis propria and the wall of the collection are penetrated. Subsequent enlargement of the fenestration is carried out (1.5–3 cm) and a nasocystic catheter may be positioned to irrigate the cavity during the following hours/days. Drainage and DEN are performed and CT and endoscopic follow up is performed. EGF was performed in 5 patients: one failed because of nonadherence between the gastric and cystic wall, while the other four patients had a rapid resolution of the WOPN within 3 weeks, with self-healing of the fenestration.

This novel technique seems promising for those collection adherents to the gastric wall; however, further studies, with a higher number of patients involved, are required to validate the EGD and better assess efficacy, costs, AEs, and long-term outcomes.

## 2. Technical Aspects

**Figure 3:**
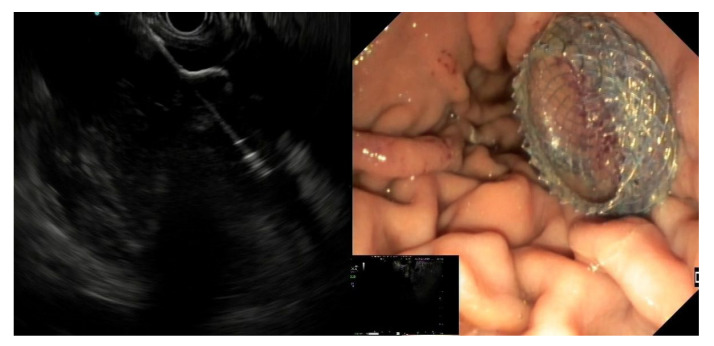
EUS image of the distal flange of an EC-LAMS deployed into a WOPN (**left**) and endoscopic view of the proximal flange after positioning the EC-LAMS (**right**).

The first preliminary step to perform DEN is the localization of the most appropriate site to practice the cystoenterostomy (usually posterior gastric or median duodenal wall) [57]. This should always be conducted under EUS assistance, since it allows one to evaluate the extent of the collection, the amount of solid vs. liquid material, thickness of the wall, and the presence of interposed structures and blood vessels (using the Doppler flow guidance) [13,41,58]. In addition, if needed, it allows one to perform FNA with subsequent culture or GRAM stain, and to inject contrast to visualize the cavity under fluoroscopy [13,41,59]. The next step is different, depending on the kind of stent used:

- DPPS: with a needle assisted procedure, a guidewire is advanced under fluoroscopic guidance and dilation of the tract is performed with an ≥8 mm pneumatic balloon [29,30]. Two or more DPPSs of 7–10 F are positioned, in order to keep the stoma open for maturation and drain the content of the collection. Furthermore, using multiple DPPSs reduces the probability of stent occlusion and migration [30,31]. Subsequent dilations with balloons of increasing diameter (15–20 mm) are performed to obtain a cystoenterostomy of caliber big enough to advance the endoscope inside the cavity and perform the DEN. If there is a need to keep the cystoenterostomy open, multiple DPPSs are placed [23,30].
- LAMS: with a needle assisted procedure, a guidewire is advanced under fluoroscopic guidance and a dilation of the tract is performed with a 4–10 mm pneumatic balloon to facilitate the advancement of the LAMS delivery catheter over the wire. The distal flange is deployed under EUS guidance, traction is then applied to appose the distal flange against the cyst wall and bring it in firm apposition against the stomach/duodenal wall. The proximal flange was then deployed under endoscopic guidance [42] or with the intra-channel release technique [60].
- EC-LAMS: the deployment catheter is positioned on the selected point on the gastric/duodenal wall. The activation of the electro-cautery tip allows direct passage of the catheter into the collection under EUS guidance. The distal flange of the LAMS is deployed, and the next steps are the same as the LAMS (Figure 3) [50].

Since the use of air instead of CO_2_ for insufflation is a well-known cause of gas embolism during DEN, current international guidelines advice to avoid air insufflation when performing DEN [13,29,61]. In fact, after its introduction in DEN practice, no gas embolism events have been reported [13].

After performing DEN, a nasocystic catheter can be placed in parallel or through the stents to cautiously flush the collection with saline or hydrogen peroxide, avoiding forced injection of high fluid volumes, which may cause rupture of the cavity wall with fatal consequences [13,30,62,63].

## 3. Devices

**Figure 4:**
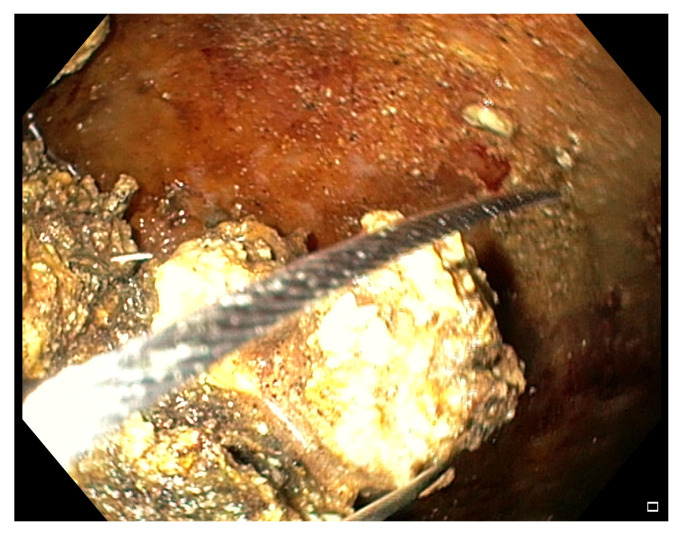
Endoscopic view of a WOPN cavity during a DEN session, where necrotic debris are grabbed with a polypectomy snare.

After entering the collection, the fluids and little necrotic debris are sucked through the endoscope working channel. The bigger necrotic debris and those adherent to the wall are grabbed with various devices conceived for other purposes, like polypectomy snares (Figure 4), Dormia basket, Roth basket, other stone removal baskets and forceps of various shape (grasping, tripod, rat-tooth, pelican), and released into the stomach/duodenum [13,23,24,25,37,41,64]. In 2018 Van Der Wiel et al. reported the first two cases of WOPN treated with a novel device, the EndoRotor Powered Endoscopic Debridement (PED) System^®^ (Interscope Medical, Inc., Worcester, MA, USA) [65].

The EndoRotor^®^ is a through-the scope tool designed for use in the gastrointestinal tract for tissue dissection and resection, later modified to perform safely and efficaciously DEN. The instrument passes through a working channel of at least 3.2 mm and has a fixed outer cannula with a hollow inner cannula that rotates at either 1000 or 1700 revolutions per minute, as set up in the electronic control console, cutting 2–4 mm of tissue per second [65,66,67]. The inner cannula has an opening through which a 50–550 mmHg negative pressure allows one to collect the cut tissue into a trap or into a standard vacuum container; both the rotating and the suction function are controlled by the operator with two separate foot pedals [66,67,68].

After two small series by Van Der Wiel et al. [67] and Larghi et al. [68], reporting a successful use of the EndoRotor^®^ to perform DEN, Stassen et al. conducted the first multicenter prospective trial involving 30 patients with symptomatic WOPN requiring DEN, whose results were recently published on the Gastrointestinal Endoscopy Journal [69]. They reported a median number of 1.5 EndoRotor^®^-assisted DENs required to achieve an average reduction of 85% in collection volume and 91% of necrotic debris. Three DEN-related AEs occurred (10% of patients), but none of them was classified as EndoRotor^®^-related (two cases of stent-related bleeding, and one case of pneumoperitoneum due to the endoscope being torqued within the LAMS), with a mortality rate of 3%.

In each series authors expressed a positive opinion, especially about the short learning curve required to easily manipulate the tool, the possibility to constantly visualize the operating area with a low estimated risk of causing complications, and the fact that no repeated retrieval and insertion of the endoscope are needed to perform the necrosectomy [65,67,68].

These data suggest that the EndoRotor^®^ may be an effective and safe dedicated device to perform DEN, but further studies are required to confront classical and EndoRotor^®^-assisted DEN.

## 4. Irrigation

Hydrogen peroxide (H_2_O_2_) is a colorless liquid which rapidly dissociates into oxygen and H_2_O when in contact with organic tissues, producing a soft foam that helps removing materials attached to the tissue, including necrotic debris [70,71]. Furthermore, it seems help wounds healing by stimulating granulation and fibrosis [72]. In order to use its healing and chemical debridement properties, it has often been used as aid in DEN, despite lack of properly designed clinical trials to assess its efficacy, AEs, and technical aspects in such field [62].

For this reasons, current international guidelines suggest restraint regarding H_2_O_2_-assisted DEN [13,14]. Many series and comparative trials, published both prior to and after the publication of the guidelines, reported a high technical and clinical success of H_2_O_2_-assisted DEN, a reduced number and duration of DEN sessions, with sometimes no need at all for mechanical debridement, and an AEs profile and rate similar to non-H_2_O_2_-assisted DEN [70,72,73,74].

Such results were confirmed by a recent systematic review and meta-analysis by Garg et al. conducted on 15 studies involving a total of 454 patients who underwent H_2_O_2_-assisted DEN (median concentration 3%, range 0.1–3% with dilution and volume 1:1–1:10 and 20 mL to 1 L respectively) [71]. Their analysis reported a high technical and clinical success rate (97.3% and 89.8% respectively), with an AEs rate of 17.9% (bleeding, stent migration, and perforation), comparable to DEN performed without H_2_O_2_.

Several cases of gas embolism have been reported after H_2_O_2_ use in neurosurgery, orthopedic surgery, fistula irrigation and wound debridement [75,76,77,78,79,80]. This potentially lethal AE may be due to the rapid and high amount of free oxygen released after the contact with organic tissue (the breakdown of 20 mL of 3% H_2_O_2_ solution releases 200 mL of gaseous oxygen) [81]. To the best of our knowledge no case of such AE has been reported in the published H_2_O_2_-assisted DEN series. Some authors suggest that H_2_O_2_-induced gas embolism may be favored by its use into closed and semi-closed cavities, where oxygen release may cause an increase of the cavity pressure, facilitating gas passage into blood vessels [72,82]. Low flow saline irrigation may be a safer alternative practice to help performing DEN.

## 5. Vacuum Assisted Closure System

It is worth mentioning that in the literature there are a few reports of the successful use of an endoscopic vacuum-assisted therapy (EVAT) and the Endo-SPONGE (B. Braun, Melsungen, Germany) for facilitating infected WOPN healing after drainage and DEN [83,84,85,86,87]. The combined action of the Endo-SPONGE (which absorbs inflammatory fluids and induces tissue granulation), together with the negative pressure (which drains inflammatory fluids, increases vascularization and cell proliferation), should lead to a faster and easier resolution of the WOPN [86,87,88]. On the other hand, this procedure is not abhorrent from risks, as highlighted by Wallstabe et al. [84]. Performing EVAT in an area rich of important vascular structures such as the region of the celiac trunk and portal venous system carries, in theory, a higher risk of bleeding then performing the therapy in other body regions [89]. As outlined in the current guidelines, the amount of series of EVAT applied to WOPN is still too small to make this technique a standard of care for PNCs [13]. Furthermore, no randomized controlled trials have been performed yet and the potential AEs profile has not yet been established.

## 6. Timing

Timing of intervention in PNCs is still a hot topic among the scientific community. Postponing any invasive intervention until the PNC has walled-off is a surgery practice heritage, which has its roots in the increased mortality and complication rate after early open necrosectomy [18,89,90,91]. Current international guidelines advise to delay any invasive intervention until at least 4 weeks after the AP onset [2]. This seems a wise approach for those patients stable enough to wait for the collection to be walled-off, but emerging data are showing that early intervention is possible and advisable for patients with clinical deterioration despite maximum support [13,14,92].

A recent survey by van Grinsven et al. reported a lack of consensus among experts regarding timing and usefulness of early PNC intervention (both drainage and necrosectomy), showing that early intervention is already incorporated into clinical practice [93]. Van Grinsven et al. conducted a randomized controlled trial (POINTER trial) with the aim of better understanding whether immediate step-up approach (endoscopic or surgical) in patients with INP is superior to postponed step-up approach with regard to clinical outcome and cost-effectiveness [19]. Results were presented at the 2020 UEG week, showing no difference between the two strategies in mortality, new onset organ failure, major complications and hospital stay. Trikudanathan et al. conducted a study on 193 patients with ANP treated with endoscopic step-up approach and/or percutaneous catheter drainage when needed [92]. Interventions were categorized as early if timing of intervention was <4 weeks (76 patients), and standard if ≥4 weeks from onset of AP. They reported an increased in mortality rate (14% vs. 4%), need for rescue open necrosectomy, hospital and ICU stay in the “early intervention” group, with similar improvement in organ failure and similar complication rate. Albers et al. treated 49 patients with confirmed or suspected IPN with STGT or MTGT with LAMS (15 mm diameter × 10 mm length or 16 mm diameter × 20 mm length) after a median of seven days after the first proof of necrosis, when most of the necrotic collections were not expected to be encapsulated [12]. DEN was always performed during the same procedure, except for patients with sepsis, where DEN was performed post-recovery. They reported a 100% technical success, an 87.8% clinical success, and an 8.2% mortality rate, in line with previous studies and meta-analysis (5.7–14.5%) [24,29,32,57].

This study also confirms results of a previous series by Yan et al., who wanted to compare the typical strategy of delaying DEN after LAMS placement (1 week), allowing maturation of the cystoenterostomy, with performing DEN immediately after LAMS positioning [58]. They found that performing DEN at the time of initial stent placement is safe, effective, and leads to an earlier WOPN clinical resolution, with similar AEs rate.

## 7. Conclusions

Symptomatic and infected necrotic collections still represent challenging and life-threatening complications of AP. Many steps have been performed after minimally invasive interventional techniques showed the first results and became available in daily clinical practice.

An endoscopic step-up approach has been proven to be, when applicable, an effective way to minimize invasiveness during interventions, reducing complications, mortality, hospital stay and total costs. When endoscopic drainage is not sufficient to gain a clinical improvement and resolution of the necrotic collection, DEN is advised in order to remove both the liquid and solid necrotic material from the collection, facilitating the healing process. When endoscopic expertise is not available, or the necrotic collection is not endoscopically reachable (when involving the paracolic gutters and/or the pelvis), or when the endoscopic approach failed to gain resolution of the necrotic collection, a surgery step-up approach is recommended [13,14].

In the last decade, many innovations and many studies allowed endoscopists to reach huge results. Diffusion of LAMS and EC-LAMS helped to simplify endoscopic maneuvers. They can be easily placed with a single step deployment platform, allowing one to effectively and safely drain necrotic collections, and immediately gain access to the cavity within a few moves. Furthermore, the availability of LAMS of different caliber (up to 20 mm), allows one to adapt the treatment to the characteristics of the wall and the content of the collection, even when composed of solid necrotic material [49]. Recent trials also demonstrated how the apposition of multiple stents in different sites, especially when dealing with large and multiloculated PNCs, facilitates the drainage of the collections, even though using LAMS reduces the need to use the MTGT when compared to PSs [50]. A recent cost-effectiveness analysis also showed how, despite being more expensive, LAMSs are more cost-effective compared to PSs [55]. Evidence, together with our experience gained over the years in collaboration with several international centers, led us to widely prefer using LAMSs over PSs for treatment of WOPN, and we think that they will be the most preferred option for end treatment of PNCs in the long term. Furthermore, in the validation of the Orlando Protocol [94], Bang et al. reported a success rate of 94.1% treating WOPN using LAMSs, compared to 84.3% using PSs.

Even if safe and effective, classical DEN is a time-consuming technique that requires multiple passages through the fistulous tract to grab necrotic debris and release them into the stomach or duodenal lumen. The EndoRotor^®^, a novel specifically designed through-the scope tool, allows one to perform DEN maintaining the endoscope into the cavity while cutting and sucking necrotic debris with the device itself, making DEN quicker and safer. An interventional randomized clinical trial is still ongoing (NCT04814693) with the purpose to better assess efficacy and safety of this device, also compared to conventional DEN [95]. New approaches and interventional techniques, like the EVAT, are under validation to reach always better technical and clinical outcomes. Clinical practice and trials showed how critical patients may benefit of an early intervention, cautiously overcoming old guidelines.

These results are drawing a path for the management of PNCs that will lead clinicians to a target approach, sewing the treatment directly on the characteristics of each patient and its disease. Hopefully, with the tremendous technological progress we are facing, in the near future newer tools and techniques will be developed. More techniques, more strategies, and more devices, mean a wider choice to reach better outcomes, reducing morbidity, mortality, and total costs, improving working conditions, and general wellbeing of the patients. In light of these rapid changes, the so-called “endoscopic step-up approach” for the treatment of WOPN is not to be intended as a linear process to follow step by step, but is more like a branched-out path where the clinical response leads to a wide range of different options about when and how to intervene. Availability of medical expertise, medical devices, and the latest medical updates will lead clinicians to the choice of the best treatment strategy possible.

More studies and more trials must take place in order to better assess what should be or become clinical daily practice, and what should be relegated to the past, as outdated by more recent data.
